# Long-Term Adverse Limb Events After Femoral Artery Endovascular Revascularization: The Boston FAROUT Study

**DOI:** 10.1016/j.jscai.2024.102241

**Published:** 2024-10-02

**Authors:** Edwin Mandieka, Ramael Ohiomoba, Piotr Sobieszczyk, Andrew C. Eisenhauer, Thomas Todoran, Scott Kinlay

**Affiliations:** a Brigham and Women’s Hospital, Boston, Massachusetts; b Harvard Medical School, Boston, Massachusetts; c Medical University of South Carolina, Charleston, South Carolina; d Ralph H. Johnson Veterans Affairs Medical Center, Charleston, South Carolina; e VA Boston Healthcare System, Boston, Massachusetts

**Keywords:** endovascular revascularization, lesion length, peripheral artery disease

## Abstract

**Background:**

: Patient, lesion, and procedural characteristics may impact the long-term risks of adverse limb outcomes differently after successful endovascular revascularization for lower extremity peripheral artery disease. The study objective was to assess the relationships of patient, lesion, and procedural characteristics to the subsequent risk of major and minor adverse limb events over the decade after successful endovascular revascularization of the superficial femoral artery for chronic limb-threatening ischemia (CLTI) or lifestyle-limiting claudication.

**Methods:**

A retrospective cohort of patients who underwent endovascular revascularization between 2003-2011 were followed for a median of 9.3 (IQR, 6.8-11.1) years. Hazard ratios (HR) and 95% CI from Cox proportional hazards models assessed the risk of major adverse limb events (MALE) (major amputation, bypass, or thrombolysis) or minor revascularization, MALE alone, and minor revascularization alone.

**Results:**

There were 232 index limb revascularizations in 185 patients. Longer lesion length was associated with a higher risk of MALE or minor revascularization (HR, 2.09; 95% CI, 1.22-3.60) and minor revascularization alone (HR, 2.53; 95% CI, 1.39-4.61). Current smoking was linked with minor revascularization (HR, 3.83; 95% CI, 1.54-9.56). CLTI was associated with MALE or minor revascularization (HR, 1.89; 95% CI, 1.09-3.29), and MALE alone (HR, 7.43; 95% CI, 3.11-17.79). Black race/ethnicity (HR, 4.74; 95% CI, 1.51-14.9) and low-density lipoprotein >100 mg/dL (HR, 2.76; 95% CI, 1.20-6.35) were linked to MALE alone.

**Conclusions:**

Factors related to MALE differed from those related to minor revascularization. Lesion length and smoking were linked to minor revascularization, whereas CLTI, Black race/ethnicity, and elevated low-density lipoprotein were linked to MALE.

## Introduction

Peripheral artery disease (PAD) affects approximately 230 million people worldwide with approximately half of patients experiencing symptoms.^1^^,^^2^ Chronic lower extremity PAD symptoms range from intermittent claudication to limb-threatening ischemia.^3^ Medical therapy that includes risk factor modification, smoking cessation, exercise therapy, and pharmacologic agents is the mainstay of therapy.^4^^,^^5^ Endovascular and surgical revascularization are indicated for chronic limb-threatening ischemia (CLTI) and to relieve lifestyle-limiting claudication nonresponsive to medical therapy.^5^

Peripheral artery disease affecting the superficial femoral artery is the most common cause of lifestyle-limiting claudication and CLTI.^6^ Although revascularization can improve symptoms, disease progression can impact quality of life, mental health, and limb function especially if it leads to adverse limb outcomes such as major amputation or the need for repeat revascularization. Revascularization also increases the risk of subsequent major adverse limb events (MALE), although this may not be a causal relationship as the need for revascularization may be a marker of more advanced disease.^7^^,^^8^

Short-term studies have shown that adverse limb outcomes are more common in non-White race/ethnicity, and patients with cigarette use, CLTI, and longer lesions.^9^, ^10^, ^11^ The long-term relationships of patient, lesion, and procedural characteristics to the risk of adverse limb outcomes after endovascular revascularization are less well known. In prior analyses, we showed that lesion length increased the risk of adverse limb events in patients with successful or failed index endovascular procedures but recurrent limb events were not related to mortality over 10 years.^12^^,^^13^ In this analysis, we focus on subjects with successful endovascular revascularization and assess the relationships of patient, lesion, and procedural characteristics to the subsequent risk of MALE or minor revascularization, MALE alone, and minor revascularization alone.

## Methods

### Study population

The Boston Femoral Artery Revascularization Outcomes (FAROUT) study was a retrospective cohort that included all patients who had their first endovascular revascularization of the superficial femoral artery for lifestyle-limiting claudication or CLTI between 2003 to 2011 at Veterans Affairs (VA) Boston or Brigham and Women’s Hospital. Patients were offered endovascular revascularization if they had lifestyle-limiting claudication after several months of medical therapy and unsupervised exercise therapy, or CLTI after disease staging by noninvasive or invasive angiography. For this analysis, we excluded unsuccessful index endovascular procedures in 21 limbs from 17 patients. After index endovascular intervention, patients were followed in the clinic at 3 months and then at 6 to 12 monthly intervals. The study was approved by the VA Boston and Brigham and Women’s Hospital Institutional Review Boards.

Subject demographics, cardiovascular risk factors, comorbidities, ankle brachial index (ABI), and medications at the time of the index procedure were extracted from the electronic medical record. Patients were defined as having CLTI if at least 1 limb had an index procedure for CLTI. Chronic kidney disease was defined as a creatinine >1.5 mg/dL, hypertension as a history of hypertension or antihypertensive medication, diabetes mellitus as a history of diabetes mellitus or treatment with diet or drug therapy, hyperlipidemia as low-density lipoprotein (LDL) >100 mg/dL or on lipid-lowering therapy, and current smoking as smoking at least 1 cigarette per day.

### Procedural variables

Procedural details were extracted from the endovascular procedure report form. These included the use of a lumen reentry device, the presence or absence of chronic total occlusion (CTO) as well as pretreatment and posttreatment stenosis. The maximum and minimum stent diameters and maximum balloon diameters were recorded, as was the success of the procedure. Angiograms in all patients were reviewed to confirm lesion length in relation to balloons and stents used as well as to record the number of tibial arteries (runoff) in the treated limb. Lesion length was stratified as <100 mm, 100 to 199 mm, or ≥200 mm. Lesion severity and extent were divided into 2 TransAtlantic Inter-Society Consensus (TASC) classifications (A or B vs C or D). Atherectomy included rotational, orbital, or laser atherectomy at the index procedure.

### Outcomes

The primary outcome of MALE or minor revascularization was assessed from the VA electronic medical record which included all presentations in the national VA health care system and Brigham and Women’s electronic medical record which included all presentations in the Mass General Brigham health care system. Outcomes were defined based on the Best Endovascular versus Best Surgical Therapy for Patients with Critical Limb Ischemia trial.^14^ MALE was defined as major amputation, new bypass graft, or thrombolysis. Outcomes were defined by 1 experienced operator (S.K.) blinded to the endovascular treatment. Minor revascularization was defined as any repeat endovascular revascularization or common femoral endarterectomy during follow-up.^12^ Limb outcomes and repeat interventions were defined for the same limb as the index procedure. In patients who had interventions in both limbs over the follow-up period, the index procedure for each limb was defined as the first procedure to that limb, and limb outcomes were recorded separately for each leg. Exploratory analyses assessed factors related to the component outcomes of MALE alone and minor revascularization alone. All outcomes were assessed up to December 2020.

### Statistical analysis

All data were analyzed using Stata version 16 (StataCorp LLC). Baseline demographics, comorbidities, and stent and balloon dimensions were described as means and standard deviations or frequencies and percentages as appropriate. Because lesion length was strongly associated with outcomes, we assessed the relationship of other lesion and procedural variables to lesion length using Fisher exact test for categorical variables and analysis of variance for continuous variables across the 3 lesion length categories. Univariable and multivariable hazard ratios (HR) and 95% CI were estimated from cause-specific Cox proportional hazards models for the relationship of patient, lesion, and procedural characteristics to MALE or minor revascularization. Additional competing risk analyses using Fine-Gray subdistribution models were used to assess the factors affecting the rate (or incidence) of MALE or minor revascularization events using death as a competing risk. In exploratory analyses, we assessed the relationship of risk of the patient, lesion, and procedural characteristics to the risk of the component end points of MALE alone, minor revascularization alone, major amputation (above the ankle), and minor amputation (below the ankle), as well as interactions between lesion length, patient, procedural and other lesion characteristics. Multivariable models used a backward stepwise process with *P* < .1 for entry for patient, lesion, and procedural variables and a *P* value to remove of >.1 with analyses clustered by the patient.

## Results

A successful index endovascular revascularization of the superficial femoral artery occurred in 232 limbs among 185 patients. Patients were followed until death or for a median of 9.3 years (IQR, 6.8-11.1 years; maximum, 15.4 years). Over the follow-up period, MALE or minor revascularization occurred in 88 (38%) of limbs. Seventy-eight (33.6%) were minor revascularization events (some limbs had multiple end points) of which 75 (30%) were repeat endovascular revascularization and 3 (1%) were common femoral endarterectomy. Twenty (8.6%) were MALE events of which 11 (4.7%) were major amputations, 9 (3.9%) were surgical bypass, and 5 (2.2%) required thrombolysis/thrombectomy. Table 1 describes the demographics, cardiovascular risk factors, comorbidities, and medications of patients at the index procedure by subsequent MALE or minor revascularization. The study population was predominantly White and male and had a high prevalence of cardiovascular risk factors and a history of coronary artery disease. There was a high penetrance of guideline-directed medical therapy. Almost all patients were taking aspirin, around 90% were on a statin, over 80% were on beta blockers, and over 60% were on angiotensin-converting enzyme inhibitors.

**Table 1:** Description of patient characteristics at the index procedure by subsequent MALE or minor revascularization in 185 subjects with successful revascularization of the superficial femoral artery.

	MALE or minor revascularization (n = 69)	No MALE or minor revascularization (n = 116)	*P* value
Age, y	66 ± 10	69 ± 10	.09
Male sex	58 (84)	82 (71)	.051
Race/ethnicity			
White	61 (88)	103 (89)	.85
Black	4 (6)	5 (4)	–
Hispanic	1 (2)	4 (3)	–
Unknown	3 (4)	4 (3)	–
Diabetes mellitus	35 (51)	56 (48)	.76
Chronic kidney disease	14 (20)	34 (29)	.23
Smoking			.004
Current	26 (38)	20 (17)	–
Former	37 (54)	73 (63)	–
Never	6 (8)	23 (20)	–
Hyperlipidemia	66 (96)	104 (90)	.18
Hypertension	66 (96)	111 (96)	1.00
History of CAD	46 (67)	87 (75)	.24
Prior CABG	26 (38)	45 (39)	1.00
Medication use			
Aspirin	67 (97)	112 (97)	1.00
Clopidogrel	57 (83)	93 (80)	.85
Statin	65 (94)	103 (89)	.30
ACE inhibitor	49 (71)	66 (57)	.06
Beta-blocker	55 (80)	97 (84)	.55
Calcium channel blocker	18 (36)	36 (31)	.85
Low-density lipoprotein >100 mg/dL	14 (20)	22 (19)	.85

Values are mean ± SD or n (%).

ACE, angiotensin-converting enzyme; CABG, coronary artery bypass grafting; CAD, coronary artery disease; MALE, major adverse limb event.

**Figure 1:**
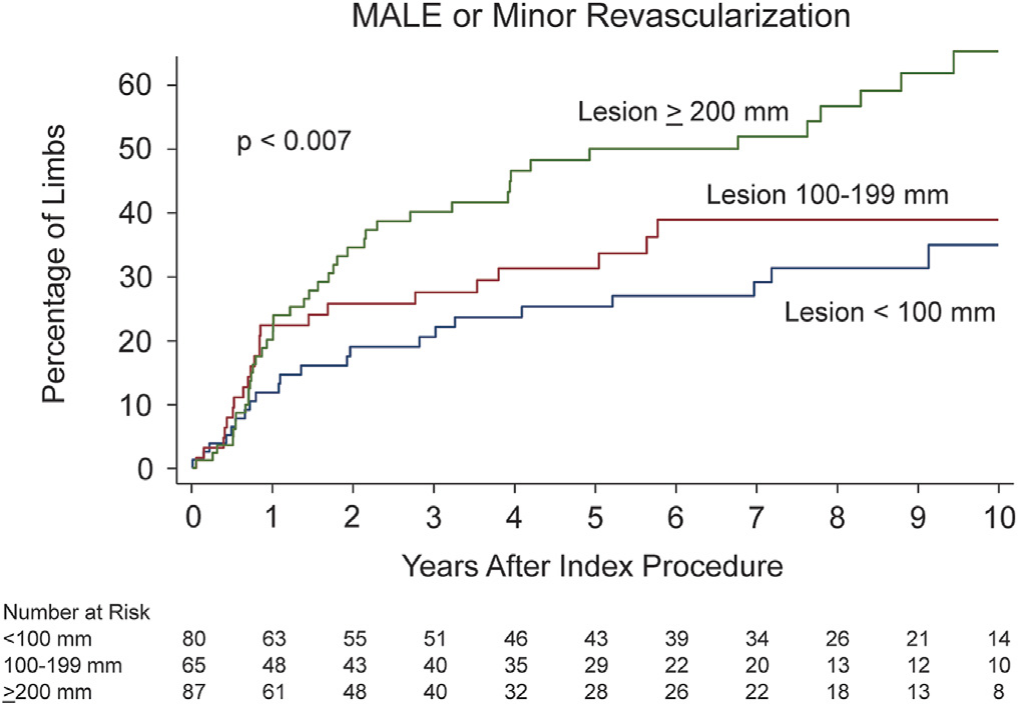
**Ten-year risk of major adverse limb event (MALE) or minor revascularization by the lesion length at the index event in 232 limbs.**

Table 2 describes the lesion and procedure characteristics among the 232 limbs with a successful index procedure and subsequent counts of MALE or minor revascularization. Prior to revascularization, the ABI was similar in limbs that did and did not subsequently have an adverse limb outcome (0.69 vs 0.68, *P* = .95). Only 3 patients had an ABI >1.4 and this was not related to adverse limb outcomes (*P* = 1.0). Patient, lesion, and procedural factors significantly related to subsequent adverse limb outcomes included lesion length, TASC lesion classification, the number of stents used, the average total length of stents, and the use of a lumen reentry device. Lesion length was the factor most strongly associated with increased risk of MALE or minor revascularization. This risk was evident in the first year after the index procedure and continued to increase over the 10 years of follow-up (Figure 1).

**Table 2:** Description of patient, lesion, and procedural characteristics at the index procedure by subsequent MALE or minor revascularization in 232 limbs with successful revascularization of the superficial femoral artery.

	MALE or minor revascularization (n = 88)	No MALE or minor revascularization (n = 144)	*P* value
Lesion length, mm			.014
<100 mm	22 ± 25	58 ± 40	–
100-199 mm	23 ± 26	42 ± 29	–
≥200 mm	43 ± 49	44 ± 31	–
Chronic total occlusion	53 (60)	71 (49)	.14
Chronic limb-threatening ischemia	17 (19)	22 (15)	.47
Preprocedure ankle brachial index	0.69 ± 0.23	0.68 ± 0.27	.95
Access			.65
Contralateral femoral	76 (86)	116 (81)	–
Other	12 (14)	28 (19)	–
TASC A or B lesion	51 (58)	60 (42)	.021
Stenting	72 (82)	101 (70)	.06
Number of stents	1.8 ± 1.3	1.4 ± 1.3	.03
Total stent length, mm	211 ± 120	172 ± 148	.04
Maximum diameter of stent or balloon, mm	6.6 ± 0.8	6.4 ± 0.9	.20
Adjunctive atherectomy	5 (6)	11 (8)	.79
Adjunctive laser	8 (9)	11 (8)	.81
Adjunctive atherectomy or laser	13 (15)	22 (15)	1.00
Adjunctive lumen reentry device	8 (9)	3 (2)	.023
Number of patent tibial arteries			.37
0-1	17 (20)	39 (27)	–
2	30 (35)	55 (39)	–
3	39 (45)	49 (34)	–

Values are mean ± SD or n (%).

MALE, major adverse limb event; TASC, TransAtlantic Inter-Society Consensus.

### Lesion length versus other factors

Table 3 shows the relationship between lesion length and procedural, patient, and other lesion variables. Longer lesion length was significantly associated with CTO, a lower preprocedural ABI, more complex TASC lesion classification (C or D), the use of stents, the number of stents, stent length, a larger stent diameter, and the use of adjunctive devices such as atherectomy, laser, and lumen reentry devices. Table 4 shows the effect of lesion length on the risk of MALE or minor revascularization for procedural, patient, and other lesion variables. After adjusting for lesion length, other lesion and procedural variables were no longer significantly related to MALE or minor revascularization indicating the predominance of this variable. Only CLTI and smoking were associated with MALE or minor revascularization after adjusting for lesion length.

**Table 3:** Description of patient, lesion, and procedural characteristics by lesion length in 232 limbs with successful revascularization of the superficial femoral artery.

	Lesion length			*P* value
	<100 mm (n = 80)	100-199 mm (n = 65)	≥200 mm (n = 87)	
Lesion length, mm	54 ± 21	132 ± 29	306 ± 67	<0.001
Chronic total occlusion	19 (24)	32 (49)	73 (84)	<0.001
Chronic limb-threatening ischemia	9 (11)	11 (17)	19 (22)	0.19
Preprocedure ankle brachial index	0.81 ± 0.28	0.67 ± 0.22	0.60 ± 0.22	<0.001
Access				0.61
Contralateral femoral	67 (84)	55 (85)	70 (81)	
Other	13 (16)	10 (15)	17 (19)	
TASC A or B lesion	80 (100)	38 (58)	3 (3)	<0.001
Stenting	48 (60)	45 (69)	80 (92)	<0.001
Number of stents	0.6 ± 0.6	1.1 ± 0.96	2.7 ± 1.1	<0.001
Average total stent length, mm	53 ± 24	135 ± 30	302 ± 79	<0.001
Maximum diameter of stent or balloon, mm	6.3 ± 1.0	6.5 ± 0.9	6.7 ± 0.7	0.02
Adjunctive atherectomy	4 (5)	8 (12)	4 (5)	0.17
Adjunctive laser	2 (3)	6 (9)	11 (13)	0.04
Adjunctive atherectomy or laser	6 (8)	14 (22)	15 (17)	0.03
Lumen reentry device	0 (0)	1 (2)	10 (11)	<0.001
Number of patent tibial arteries				0.99
0-1	18 (23)	18 (28)	20 (23)	
2	30 (38)	23 (35)	32 (38)	
3	31 (39)	24 (37)	33 (39)	

Values are mean ± SD or n (%).

TASC, TransAtlantic Inter-Society Consensus.

**Table 4:** Hazard ratios and 95% CI for patient, lesion, and procedural characteristics and subsequent MALE or minor revascularization from univariable Cox proportional hazards models and models with adjustment for lesion length.

	Univariable HR (95% CI)	Adjusting for length HR (95% CI)
Lesion length		
<100 mm	1 (reference)	
100-199 mm	1.40 (0.78-2.53)	
≥200 mm	2.19 (1.28-3.77)^a^	
Chronic limb-threatening ischemia vs claudication	2.01 (1.15-3.51)^b^	1.84 (1.06-3.20)^b^
Smoking status		
Never smoked	1 (reference)	1 (reference)
Former smoker	1.92 (0.90-4.10)	1.87 (0.89-3.92)
Current smoker	2.98 (1.37-6.51)^a^	2.97 (1.39-6.37)^a^
Chronic total occlusion	1.47 (0.95-2.28)	1.05 (0.63-1.74)
Preprocedural ankle brachial index	1.09 (0.36-3.28)	1.73 (0.57-5.24)
Contralateral femoral access	1.14 (0.62-2.10)	1.81 (0.68-2.06)
TASC A or B vs C or D	1.79 (1.15-2.78)^b^	1.09 (0.51-2.32)
Stenting	1.51 (0.84-2.70)	1.21 (0.67-2.17)
Number of stents	1.17 (1.01-1.36)^b^	0.99 (0.82-1.20)
Maximum diameter of stents or balloons	1.12 (0.86-1.45)	1.03 (0.79-1.36)
Atherectomy	0.80 (0.34-1.88)	0.81 (0.35-1.91)
Laser	1.10 (0.46-2.63)	0.88 (0.36-2.19)
Atherectomy or laser	0.96 (0.51-1.80)	0.85 (0.44-1.62)
Lumen reentry devices	2.34 (1.29-3.89)^a^	1.61 (0.88-2.96)
Number of patent tibial arteries	1.07 (0.82-1.39)	1.04 (0.80-1.35)

MALE, major adverse limb event; TASC, TransAtlantic Inter-Society Consensus.

a *P* < .01,

b *P* < .05.

**Figure 2:**
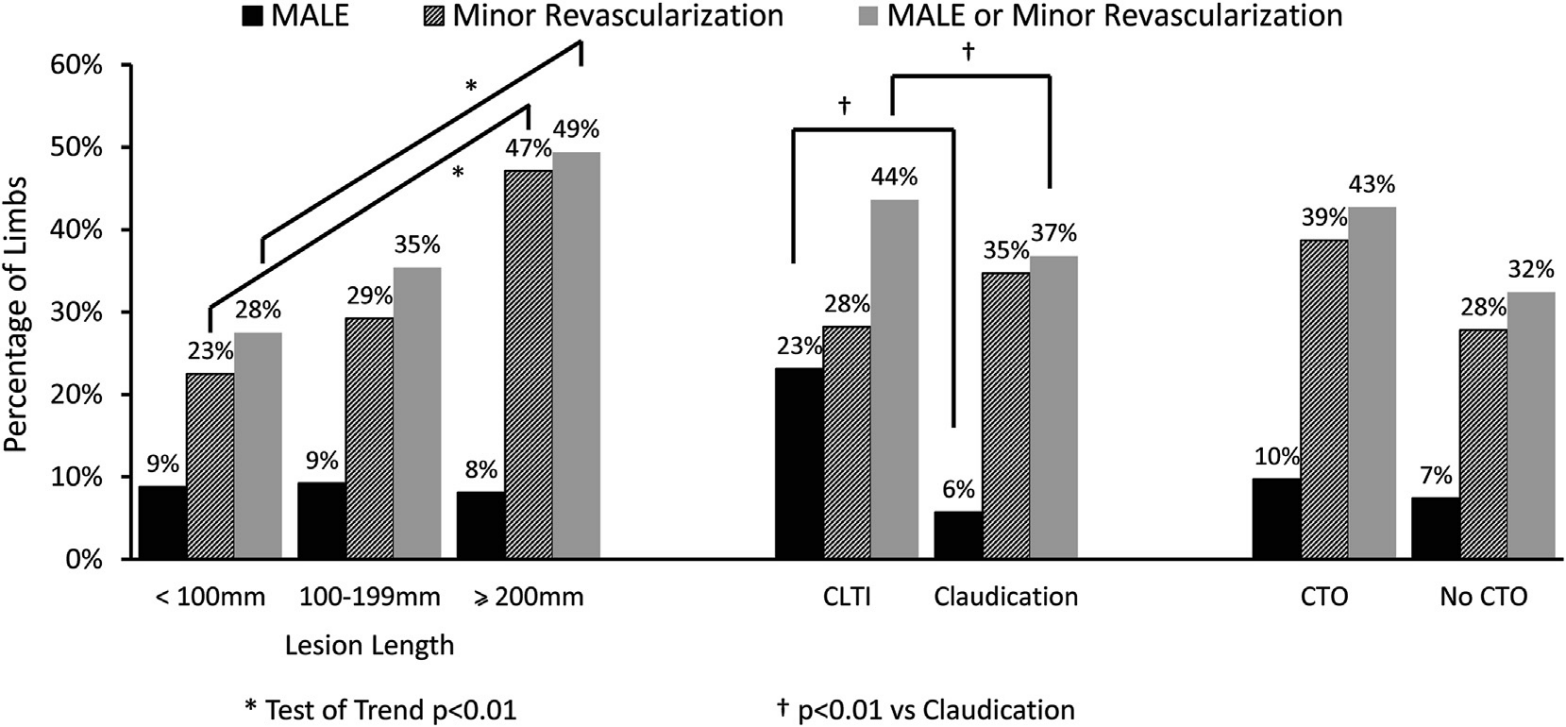
**Relationship of lesion length, chronic limb-threatening ischemia (CLTI) versus claudication, and the presence or absence of a chronic total occlusion (CTO) on the subsequent risk of major adverse limb event (MALE), minor revascularization, and MALE or minor revascularization.**

In exploratory analyses, we assessed the relationship of lesion length, CLTI, and the presence or absence of a CTO at the index event with subsequent component outcomes of MALE alone and minor revascularization alone (Figure 2). Increasing lesion length was significantly associated with minor revascularization, but not MALE. However, compared to claudication at the index procedure, CLTI was significantly related to MALE but not minor revascularization (Figure 2). CTO at the index procedure was not significantly related to any outcome.

### Lesion and patient factors related to adverse limb outcomes

In Table 5, the final multivariable models for MALE or minor revascularization, MALE alone, and minor revascularization alone show substantial differences in the factors related to the combined end point compared to the component end points. Lesion length and smoking primarily affected the risk of minor revascularization, which was the larger component of the combined end point of MALE or minor revascularization. In contrast, CLTI, Black race/ethnicity, and elevated LDL cholesterol >100 mg/dL were related to subsequent MALE alone (Central Illustration). Table 6 shows the final multivariable models in competing risk models adjusting for the competing risk of death. The results are the same as for the HR from the cause-specific Cox proportional hazards models in Table 5.

**Table 5:** Final multivariable models for MALE or minor revascularization, MALE alone, and minor revascularization alone with HR and 95% CI from Cox proportional hazards models.

Variable	MALE or minor revascularization HR (95% CI)	MALE HR (95% CI)	Minor revascularization HR (95% CI)
Lesion length			
<100 mm	1 (reference)	–	1 (reference)
100-199 mm	1.53 (0.86-2.73)	–	1.47 (0.75-2.87)
≥200 mm	2.09 (1.22-3.60)^a^	–	2.53 (1.39-4.61)^a^
Chronic limb-threatening ischemia vs claudication	1.89 (1.09-3.29)^b^	7.43 (3.11-17.79)^a^	–
Smoking status			
Never smoked	1 (reference)	–	1 (reference)
Former smoker	1.87 (0.89-3.91)	–	2.94 (1.23-7.06)^b^
Current smoker	3.03 (1.43-6.43)^a^	–	3.83 (1.54-9.56)^a^
Race/Ethnicity			
White	–	1 (reference)	–
Hispanic or Other	–	2.39 (0.51-11.12)	–
Black	–	4.74 (1.51-14.9)^a^	–
Low-density lipoprotein > 100 mg/dL	–	2.76 (1.20-6.35)^b^	–

HR, hazard ratio; MALE, major adverse limb event.

a *P* < .01,

b *P* < .05.

**Central Illustration:**
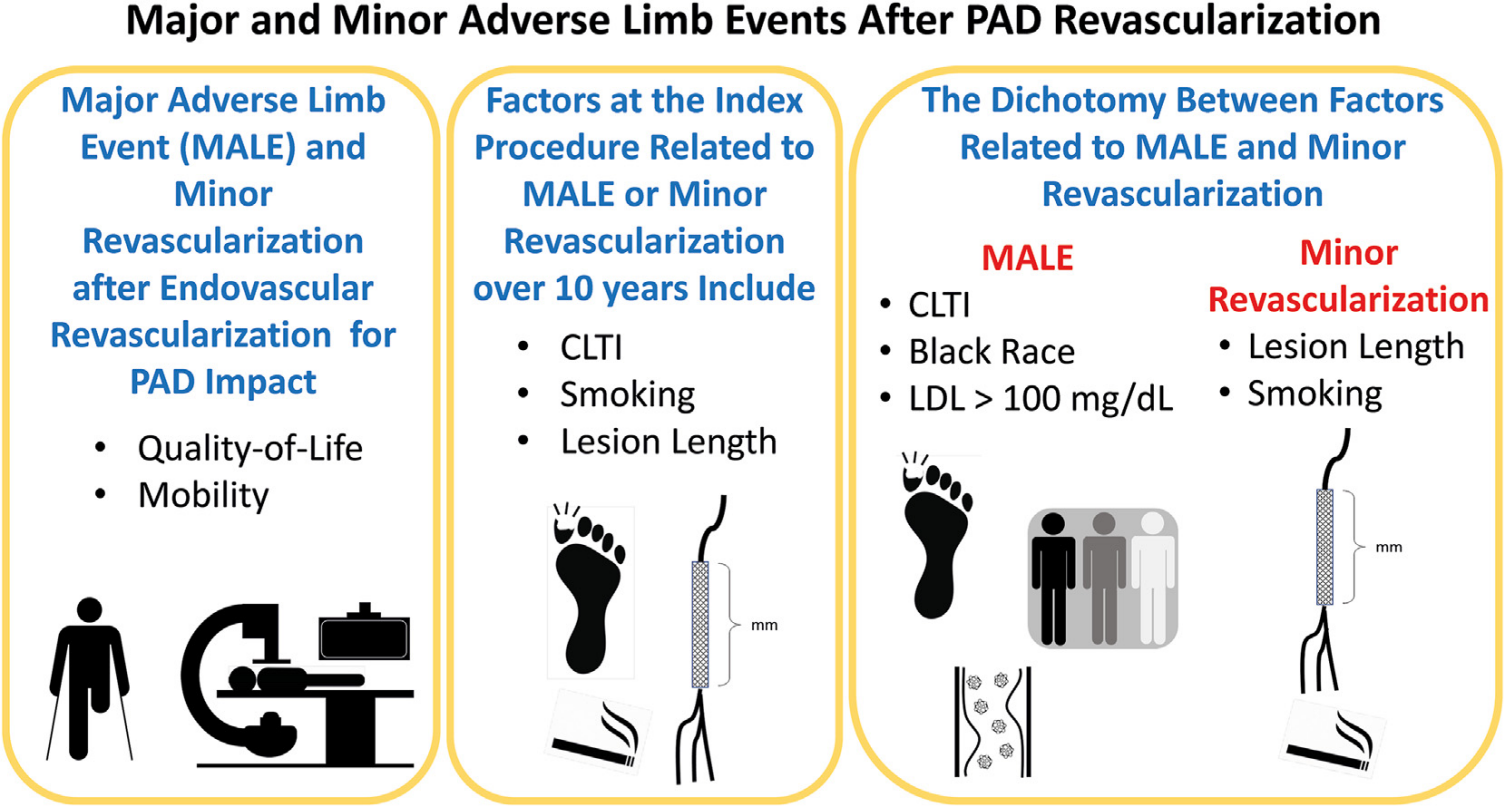
Patient and lesion factors related to major adverse limb event (MALE), minor revascularization, or both end points combined. CLTI, chronic limb-threatening ischemia; LDL, low-density lipoprotein; PAD, peripheral artery disease.

**Table 6:** Final competing risk multivariable models for MALE or minor revascularization, MALE alone, and minor revascularization alone with SHR and 95% CI from Fine-Gray subdistribution models with a competing outcome of death.

Variable	MALE or minor revascularization SHR (95% CI)	MALE SHR (95% CI)	Minor revascularization SHR (95% CI)
Lesion length			
<100 mm	1 (reference)	–	1 (reference)
100-199 mm	1.49 (0.84-2.64)	–	1.41 (0.73-2.73)
≥200 mm	1.96 (1.49-3.35)^a^	–	2.24 (1.24-4.06)^a^
Chronic limb-threatening ischemia vs claudication	–	4.23 (1.74, 10.34)^a^	–
Smoking status			
Never smoked	1 (reference)	–	1 (reference)
Former smoker	1.49 (0.71-3.10)	–	2.42 (1.0-5.87)
Current smoker	2.66 (1.25-5.68)^a^	–	3.51 (1.39-8.84)^a^
Race/ethnicity			
White	–	1 (reference)	–
Hispanic or other	–	2.19 (0.46-10.42)	–
Black	–	4.58 (1.45-14.39)^a^	–
Low-density lipoprotein > 100 mg/dL	–	3.10 (1.31-.33)^b^	–

MALE, major adverse limb event; SHR, subdistribution hazard ratio.

a *P* < .01,

b *P* < .05.

### Amputations

There were 11 major amputations and 8 minor amputations over the follow-up period. Amputations were significantly higher in patients with CLTI at the index procedure (HR, 11.12; 95% CI, 4.27-28.93; *P* < .001), in Black patients (HR, 6.64; 95% CI, 2.60-16.96; *P* < .001), and patients with an LDL greater than 100 mg/dL (HR, 2.51; 95% CI, 1.07-5.93; *P* = .035).

## Discussion

In this cohort of patients who underwent successful endovascular revascularization of the superficial femoral artery, we found that lesion length, particularly lesion length ≥200 mm, was the dominant procedural factor related to MALE or minor revascularization and this was driven by its strong relationship to minor revascularization. This risk occurred in the first year and continued to accumulate over the 10 years of follow-up. Lesion length was strongly associated with several other procedural and lesion characteristics such as CTO, TASC classification, stent use, stent length, and adjunctive devices such as atherectomy and lumen reentry devices. Some of these relationships are intuitive as stents are more often used in long lesions and reentry devices for some CTO. However, after adjusting for lesion length, these other procedural and lesion characteristics were no longer related to adverse limb outcomes.

The relationship of lesion length to minor revascularization is likely driven by its relationship to restenosis. Other smaller cohorts with 1 to 3 years of follow-up have identified lesion length as an important factor in determining the short-term risk of restenosis after femoral artery stenting.^15^, ^16^, ^17^ Our study shows that this early risk is magnified over a much longer time frame of 10 years after the index procedure, and suggests that surveillance for restenosis and recurrent symptoms is particularly important in patients who have endovascular interventions for long lesions.

In contrast to the relationship between lesion length and minor revascularization, we found CLTI was associated with the risk of MALE or minor revascularization, and this was driven by MALE alone. Studies with shorter follow-ups have documented the increased risk of MALE with CLTI^18^ but were not able to unravel the dichotomy between CLTI and MALE versus lesion length and minor revascularization as we have done in our analysis. This suggests that these 2 factors contribute to the overall risk of MALE or minor revascularization through different pathways. Concordant with available data, our analysis showed CLTI was associated with an increased risk of any amputation.^19^ CTO did not affect the risk of any component outcome, nonetheless, we only focused on successful index procedures, and this variable is more closely related to index revascularization success.^10^

Further exploratory analyses showed that 2 demographic variables, Black race/ethnicity, and LDL >100 mg/dL were related to a higher risk of MALE but not minor revascularization. These factors were also strongly related to the risk of major or minor amputations. The higher long-term risk of MALE related to Black race/ethnicity is reflected in several short-term population studies showing higher rates of nontraumatic amputation among Black, Hispanic, and Native American people after PAD revascularization.^20^^,^^21^ Our study highlights the continued need to tackle PAD disparities by increasing clinician awareness of health disparities, diversifying the clinician workforce, improving access to high-quality health care, community collaborations, health policy that addresses social determinants of health, and robust inclusive research.^11^^,^^22^

Although virtually all patients were on statin therapy, we found an increased risk of MALE alone and any amputation with elevated LDL cholesterol but not minor revascularization suggesting intensive LDL lowering may be more important for preventing atherosclerosis progression causing MALE. This is supported by a substudy of the Further Cardiovascular Outcomes Research with PCSK9 Inhibition in Subjects with Elevated Risk trial, where intensive LDL lowering with a PCSK9 inhibitor decreased the risk of MALE, but not any peripheral revascularization compared to placebo.^23^

Furthermore, our study underscores the importance of smoking cessation in patients with PAD. Active cigarette smoking was strongly related to MALE or minor revascularization. This was driven by a very strong relationship to minor revascularization, which points to the importance of smoking in determining restenosis and potentially new lesions. Our exploratory analysis showed that former smoking also increased the risk of repeat endovascular revascularization and suggests that current and former smokers need closer surveillance for recurrent disease than patients who have never smoked.

Competing risk analyses adjusting for the competing risk of death showed similar results. Cox proportional hazards models are often interpreted as assessing factors related to the etiology of an outcome (in this case adverse limb outcomes), whereas competing risk analyses provide more information on the rate or likelihood that a patient will experience an adverse limb outcome, given that some patients may die before they can experience an outcome.^24^

Lastly, newer therapies particularly drug-eluting stents and drug-coated balloons have been shown to be more effective in the treatment of long lesions through higher patency rates and freedom from revascularization.^25^^,^^26^ However, they do not decrease the risk of MALE.^27^ This supports the concept raised in our study that precipitants and therapies for restenosis may be different from those for MALE.

### Limitations

Our study had low representation of women and minorities largely reflecting the Veteran population in the North East of the United States.^12^ Nevertheless, we were able to discern important differences in race and ethnicity that reflect other studies of health disparities in PAD.^28^ These concerns justify the need for system-based interventions, physician workforce interventions, and community education models to mitigate PAD disparities.^28^ Although our study may have been smaller than other observational studies, the longer follow-up increased the power to find differences and provided novel information over this very long period. We were not able to assess the impact of supervised exercise therapy which was not approved during most of the follow-up time. Lastly, our cohort preceded the newer generation stent, balloons, and pharmacotherapies such as direct oral anticoagulants and proprotein convertase subtilisin/kexin type 9 inhibitors which have shown lower rates of adverse limb events.^29^, ^30^, ^31^, ^32^

## Conclusion

Lesion length ≥200 mm is the dominant lesion factor for future risk of MALE or minor revascularization after successful endovascular revascularization for superficial femoral artery PAD. Additional factors related to this outcome are CLTI and smoking. The dichotomy between risk factors for MALE alone and minor revascularization alone was a novel finding. CLTI, Black race/ethnicity, and LDL cholesterol >100 mg/dL were associated with an increased risk of both MALE and amputation. Lesion length and cigarette smoking were related to minor revascularization. These factors identify different targets to decrease MALE and minor revascularization for the overall health of patients after a successful endovascular revascularization of the superficial femoral artery.
